# Trauma and Injuries Pattern During Hajj, 1443 (2022): A Cross-Sectional Study

**DOI:** 10.7759/cureus.41751

**Published:** 2023-07-12

**Authors:** Majed M Al-Hayani, Shady Kamel, Ahmad M Al-Hayani, Emad A Al-Hazmi, Mervat S Al-Shanbari, Noha S Al-Otaibi, Abdulaziz S Almeshal, Abdullah M Assiri

**Affiliations:** 1 Field Epidemiology Training Program, Ministry of Health, Riyadh, SAU; 2 College of Medicine, King Abdulaziz University, Rabigh, SAU; 3 Field Epidemiology Training Program, Ministry of Health, Makkah, SAU; 4 College of Medicine, Al-Sibai Health Institute, Jeddah, SAU; 5 Nursing, International Academy of Health Sciences, Makkah, SAU; 6 Deputyship of Public Health, Ministry of Health, Riyadh, SAU

**Keywords:** accidental injuries, accidental falls, injury, trauma and injury prevention, public health system, mass gathering medicine, hajj, trauma

## Abstract

Introduction

Trauma and injuries are common among pilgrims during Hajj, the biggest mass gathering event. Trauma and injury causes vary from falling and pressing in crowds to being burned by boiled water and road traffic accidents (RTA). Time to reach the hospital during highly condensed areas in Hajj are challenges for the public health authorities and the healthcare system to achieve optimum control, management, and outcome. This study aims to explore the pattern of trauma and injuries during Hajj as it is crucial to improve future preventive measures and care quality.

Methods

A cross-sectional questionnaire-based study was conducted in one hospital in each of the Mena and Arafat (Al-Mashaar's areas) in Makkah City, Saudi Arabia, from July 8 to 10, 2022. Data was collected through interviews with patients who visit the hospitals or enter the emergency department and are diagnosed with trauma or injury during the Hajj season of 1443 Hijri date (2022).

Results

A total of 264 people volunteered to participate in the survey. The mean age by years was 43.5 ± 10.7, and the majority (56%) were between 41 and 64. There were multiple nationalities - the most common nationality was Egyptian (25%), followed by Saudi (10%). The commonest type of trauma was cutting wounds (50%), and the commonest cause was falling (39%), followed by foot twisting (31%). There were 142 cases in Arafat and 122 cases in Mena in the study duration. Tissue contusions are higher in Arafat. Fractures (5%) were in both areas but higher in Mena with burns and sprains. Friction blister injuries were only in Mena and were statistically associated with walking barefoot (p<0.01), which was associated with Egyptians (p<0.05). Also, thigh chafing is only in Mena, while eye traumas and abrasion are only in Arafat. There were four causes of injury that are statistically significantly associated with the area (p<0.05): foot twisting in Arafat, pressing in overcrowding, stoning, and burning in Mena. Moreover, all the RTA cases (n=4) were in Arafat, and all the stoning and burning by boiling water were in Mena. Admission was only for burning (n=2) and falling (n=2) cases and only in Mena emergency hospital; otherwise, all trauma cases were discharged after receiving management - no deaths among the study sample. Injuries in Mena are likely to happen in the evening and night (n=91), while in Arafat, it is more likely in two periods (n=113), in the early morning and afternoon. This difference is statistically significant between the two areas (p<0.05). Most pilgrims (n=129/253) reach the hospital in 16 to 30 minutes. A statistically significant association exists between the duration and the area (p<0.05). Most patients in Arafat (88%) reach the hospital in less than 30 minutes, while only 50% take the same duration in Mena.

Conclusion

The Hajj season of 1443 H (2022) has a similar trauma pattern and improved outcomes compared to previous seasons. Discovering and digging into the causes of traumas and injuries should be optimized in future research for better control and customized prevention measures. Establishing new and remodeling current prevention measures is recommended for more control.

## Introduction

Trauma and injuries are common among pilgrims during Hajj, the biggest mass gathering event worldwide held every Hijri year in the Kingdom of Saudi Arabia (KSA) [1]. Trauma and injury causes are varied from falling while walking in a crowd and burning boiled water to road traffic accidents [2]. The movement of huge numbers of pilgrims in one area and roads with limited width greatly increases the risk of injuries [3].

Time to reach the hospital and triaged in the emergency room during highly condensed areas and busy staff in Hajj are challenges for the healthcare system. Delays in transportation lead to more harm to the patient and increase the load and care time in the health facility [4]. Moreover, controlling and preventing trauma before they occur is possible and contributes to improving the service provided [5]. Investigation of the causes of trauma during hajj and understanding the pattern and the trends of trauma is crucial to implementing better measures.

There is a difference between health conditions in general between the Mena area and the Arafat area, as well as in the rituals performed in each of them, with common factors that lead to traumas, such as walking in crowdedness and traffic road accidents [1]. Pilgrims move to Arafat in one day (the ninth day of Thul Hijja) from the morning to the evening and then move out to another area, while in Mena, they stay there from day eight and back to it on the night of the ninth day or just before the sunset of the tenth of Thul Hijja to start the rituals of Mena. Studying and finding differences may lead to harmonizing experiences in dealing with and preventing injuries in the coming seasons of Hajj. Furthermore, some chronic diseases have a higher risk of developing complications with trauma [6]. Studying the trauma outcome will improve the control measure for these specific groups of pilgrims.

In this study, we will explore the frequency and pattern of trauma and received management during Hajj in Al-Mashaar areas and investigate the difference in trauma patterns between the areas. All this is to find out the strengths and weaknesses of the health system in dealing with trauma during Hajj and, more importantly, the education program before Hajj season starts.

## Materials and methods

The study was approved to be conducted by The Global Centre for Mass Gatherings Medicine, Ministry of Health, Riyadh, KSA. Ethical approval was obtained from the institutional review board in King Fahad Medical City, Riyadh, Saudi Arabia (reference number: 23-273E).

Study type, population, and sampling

We conducted a cross-sectional questionnaires-based study among the pilgrims during the Hajj season of 1443 Hijri date (2022). The research team filled out the questionnaires through interviews with patients, from July 8 to 10, 2022, in one hospital in each area of Masha'er: Arafat and Mena in Makkah City, KSA. The targeted population is all the pilgrims who visit hospitals with a chief complaint of trauma or injury. We used the convenience sampling method as the research team was in the hospitals at a specific time when pilgrims were performing their rituals in the targeted area.

Data collection

Basic demographic data and current history of trauma were collected using a questionnaire and through interviews with the patient after verbal informed consent to ensure voluntary participation. The interview is established only after confirming the diagnosis and whether they were in the emergency room, clinic, or waiting area.

The questionnaires filled by authors during an interview with the patients in two hospitals in two regions (Masha'er): Arafa and Mena, start from July 8 (Thul Hijja 9) to July 10 (Thul Hijja 11), 2022. Only trauma patients who visited trauma clinics or were triaged to emergency trauma beds are eligible to participate in the study.

The questionnaire consisted of three parts. The first part of the questionnaire included the following socio-demographical data: age, gender, nationality, and comorbidities. The second part is about how the patient came to the hospital, the duration he took, and the management outcome (discharge, admission, or death). The third part included questions regarding the trauma time, type, site, and cause.

We include patients above 16 years old with a confirmed diagnosis of trauma/accidental injury during the period of Hajj. We exclude patients of pediatric age (16 years and lower), non-pilgrim (hospital staff, organizers, security staff, and others), and patients with trauma or injury onset before Hajj season.

Data analysis

Descriptive results will be presented as numbers and proportions. A chi-square and Fisher exact tests will assess the association between demographics, trauma characteristics, time, and transport. Missing data is excluded from the analysis. Statistical Package for Social Sciences (SPSS) software version 25.0 (SPSS IS Inc., Chicago, IL, US) will be used for all data analyses. A two-tailed p-value of < 0.05 will be considered significant.

## Results

**Figure 1:**
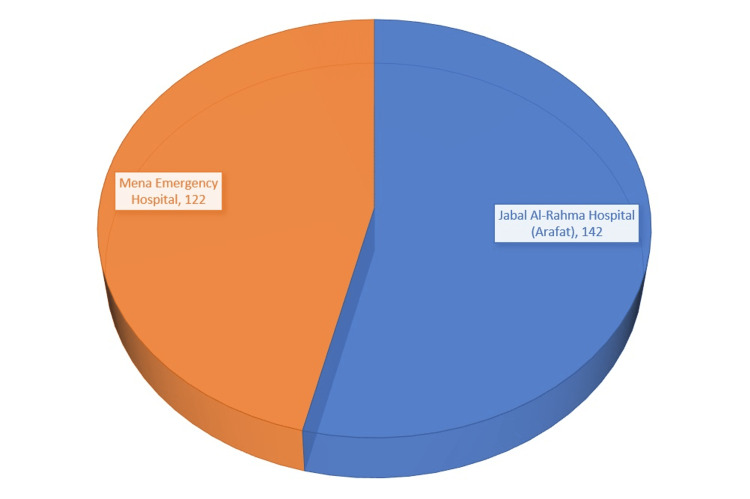
Distribution of trauma cases by number during Hajj 1443 H in two hospitals (areas) in Makkah, Saudi Arabia. H: Hijri date

A total of 264 people volunteered to participate in the survey. Figure 1 shows the number of cases in each hospital of both areas (Mena and Arafat) of the study.

Demographic data

Of the participants, the majority (51%) are between 33 and 48 years old, and approximately 2% are old (65 and above). Almost three-quarters of the sample are male (73%). Like the total demographics, males are above 70% in both areas. There were 29 nationalities, and it was unknown in eight cases. The most common nationality is Egyptian (25%), and then by quite a bit the Saudi (10%), Algeria (8%), Pakistani (7%), Morocco (6%), and Syrian (5%) followed by the other nationalities. Egyptians are the most common among nationalities in both areas, followed by Saudis in Arafat (n=17) and Pakistanis in Mena (n=13). Sixty-eight of the sample have comorbid chronic disease. The most common was diabetes mellites (74%), followed by bronchial asthma (24%). Detailed demographic data is displayed in Table 1.

**Table 1:** Demographics of trauma patients in two areas during Hajj 1443 H in Makkah, Saudi Arabia. *Four missing data, **Nine missing data, ***Three missing data H: Hijri date

Variable	Total	Mena	Arafat
	n (%)		
Age by years*			
mean ± SD (range)	43.51 ± 10.66 (17-79)	45.09 ± 10.79 (18-79)	41.68 ± 10.20 (17-68)
17-32	41 (15.77)	21 (17.50)	20 (14.29)
33-48	133 (51.15)	71 (59.17)	62 (44.29)
49-64	81 (31.15)	27 (22.50	54 (38.57)
65-80	5 (1.92)	1 (0.83)	4 (2.86)
Gender**			
Male	194 (73.48)	93 (76.86)	101 (75.37)
Female	61 (26.52)	28 (23.14)	33 (24.63)
Nationality***			
Egyptian	67 (25.38)	32 (26.23)	35 (24.65)
Saudi	25 (9.47)	8 (6.56)	17 (11.97)
Algeria	21 (7.95)	8 (6.56)	13 (9.15)
Pakistani	19 (7.20)	13 (10.66)	6 (4.23)
Morocco	17 (6.44)	7 (5.74)	10 (7.04)
Syrian	14 (5.30)	7 (5.74)	7 (4.93)
Other	96 (36.36)	47 (38.52)	54 (38.03)
Comorbidity			
Have at least one comorbidity	68 (25.76)	37 (30.33)	31 (21.53)
Diabetes mellites	50 (18.94)	24 (19.67)	26 (18.06)
Bronchial asthma	16 (6.06)	11 (9.02)	5 (3.47)
Hypertension	8 (3.03)	8 (6.56)	0 (0)
Heart disease	9 (3.41)	6 (4.92)	3 (2.08)
Other	9 (3.41)	6 (4.92)	3 (2.08)

Transportation

As shown in Table 2, in Mena, the injuries are likely to happen in the evening and night (n=91), while in Arafat, it is higher in two periods (n=113), in the early morning and afternoon. This difference is statistically significant between the two areas (p<0.05).

**Table 2:** Trauma frequency by time interval and duration of transport to the hospital in Mena and Arafat during Hajj 1443 H in Makkah, Saudi Arabia. *Indicates a significant difference H: Hijri date

Variable	Mena	Arafat	p-value
	n (%)		
Time intervals			
Early in the day (12 am to 6 am)	7 (5.74)	66 (46.48)	0.000*
Late in the day (6 am to 12 pm)	7 (5.74)	24 (16.90)	
Afternoon (12 pm to 6 pm)	3 (2.46)	47 (33.10)	
Evening and night (6 pm to 12 am)	91 (74.59)	0 (0)	
Unknown	11 (9.02)	5 (3.52)	
Duration of transport			
15 minutes and less	10 (8.20)	47 (33.10)	0.000*
16-30 minutes	51 (41.80)	78 (54.93)	
31-45 minutes	23 (18.85)	7 (4.93)	
46-60 minutes	16 (13.11)	0 (0)	
More than one hour	20 (16.39)	3 (2.11)	
Unknown	2 (1.64)	7 (4.93)	

Most pilgrims (n= 129/253) reach the hospital in 16 to 30 minutes. There is a statistically significant association between the duration and the area (p<0.05) as most of the patients in Arafat reach the hospital in a maximum of 30 minutes, while in Mena, most of them (n=51) reach the hospital in 16 to 30 minutes but many (n=59) also reach the hospital in more than 30 minutes and until more than one hour.

Table 3 shows that the majority came to the hospitals by walking (78%) and the duration of 30 minutes or less (69%), but 83% of those who took more than 60 minutes came walking (n=19/23). Ambulance users (n=8) reach the hospital in 30 minutes or less. It is worth mentioning that only two of the ambulance users (n=2/8) were road traffic accidents (RTA) cases (n=2/4), and the other were falling cases, foot twisting, and pressed in overcrowdings.

**Table 3:** Duration to reach the hospital by the used method of transportation during days 9 to 11 of Hajj 1443 H in Mena and Arafat in Makkah, Saudi Arabia. H: Hijri date

Variable		Method of transport to hospital				
		Walking	Wheelchair	Ambulance	Golf car	Unknown
Duration of transport	1-15 min	42 (20.59)	12 (25)	3 (37.50)	0 (0)	0 (0)
	16-30 min	98 (48.04)	25 (52.08)	5 (62.50)	0 (0)	1 (33.33)
	31-45 min	23 (11.27)	6 (12.5)	0 (0)	0 (0)	1 (33.33)
	46-60 min	14 (6.86)	2 (4.17)	0 (0)	0 (0)	0 (0)
	> 60 min	19 (9.31)	3 (6.25)	0 (0)	1 (100)	0 (0)
	Unknown	8 (3.92)	0 (0)	0 (0)	0 (0)	1 (33.33)
Total		204 (100)	48 (100)	8 (100)	1 (100)	3 (100)

Trauma type, site, cause, and outcome

**Figure 2:**
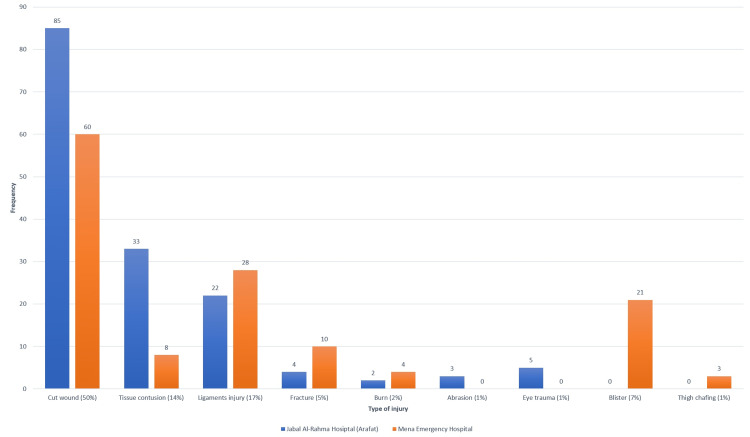
Distribution of injury types during Hajj 1443 H in two hospitals (areas) in Makkah, Saudi Arabia. H: Hijri date

Figure 2 shows the type of traumas in both areas. Cuts wounds (50%) are the highest in both areas. Fractures, burns, and sprains (ligament injury) are higher in Mena, while in Arafat, tissue contusions. Blisters and thigh chafing are only in Mena, while eye traumas and abrasion are only in Arafat. It must mention that the cause of burning in Arafat is sunburn, while in Mena due to boiling water.

Regarding the site of trauma, in both areas, the lower limb was the most affected (n=233/264), and the foot was the most affected in the lower limb (n=177/233). Excepting the lower limb, in Arafat, there was more trauma in the head (67%) and upper limb (63%) (except the hand, which was equal to Mena cases). In other words, the upper limb was more affected in the Arafat area and the lower limb in both areas. In Mena, there was more trauma in the chest (73%) and abdomen (70%). There is no statistically significant association between the site of trauma and the area, except for the abdomen (p<0.01) (Table 4). The abdomen is associated significantly when the cause of the trauma is burning by boiling water (n=4/4) (p=0.000), RTA (n=3/4) (p=0.005), falling (n=21/126) (p=0.009), and foot twisting or ankle sprain (n=6/98) (p=0.039).

**Table 4:** Trauma site during Hajj 1443 H in Mena and Arafat areas in Makkah, Saudi Arabia. *Indicates a significant difference H: Hijri date

Site	Total	Mena	Arafat	p-value
	n (%)			
Lower limb	233 (73.04)	112 (48.07)	121 (51.93)	
Foot	177 (55.49)	87 (49.15)	90 (50.85)	0.172
Leg	12 (3.76)	6 (50)	6 (50)	0.788
Thigh	44 (13.79)	19 (43.18)	25 (56.82)	0.659
Abdomen	30 (9.40)	21 (70)	9 (30)	0.006*
Chest	11 (3.45)	8 (72.73)	3 (27.27)	0.072
Head	21 (6.58)	7 (33.33)	14 (66.67)	0.217
Upper limb	24 (7.52)	9 (37.50)	15 (62.50)	
Arm	3 (0.94)	0 (0)	3 (100)	0.106
Forearm	5 (1.57)	1 (20)	4 (80)	0.235
Hand	16 (5.02)	8 (50)	8 (50)	0.754
Total	319 (100)	166 (52.04)	153 (47.96)	

The commonest cause of the trauma is falling (39%), followed by foot twisting (31%) and pressing in overcrowding (17%). Walking barefoot (100%) and walking only (78%) are both common in Mena. Additionally, most blisters are caused by walking barefoot (86%). None of the diabetic patients were walking barefoot. Furthermore, the other four causes that are statistically significant (p<0.05) associated with the area are pressing in overcrowding, foot twisting or ankle sprain, stoning, and burning. Moreover, all the RTA cases were in Arafat, and all the stoning was in Mena (Table 5).

**Table 5:** Trauma causes in Mena and Arafat areas during Hajj 1443 H in Makkah, Saudi Arabia. *Indicates a significant difference H: Hijri date

Cause	Total	Mena	Arafat	p-value
	n (%)			
Pressing in overcrowding	55 (17.13)	38 (69.09)	17 (30.91)	0.000*
Foot twisting/ankle sprain	98 (30.53)	36 (36.73)	62 (63.27)	0.018*
Falling	126 (39.25)	57 (45.24)	69 (54.76)	0.762
Walking	9 (2.80)	7 (77.78)	2 (22.22)	0.053
Walking barefoot	19 (5.92)	19 (100)	0 (0)	0.000*
Stoning	4 (1.25)	0 (0)	4 (100)	0.044*
Burning by boiling water	4 (1.25)	4 (100)	0 (0)	0.044*
Road traffic accident (RTA)	4 (1.25)	0 (0)	4 (100)	0.062
Other	2 (0.62)	2 (100)	0 (0)	0.212
Total	321 (100)	167 (52.02)	154 (47.98)	

It is worth mentioning that Egyptian nationality has a statistically significant association with walking barefoot (p=0.039), while Saudi nationality is associated with foot twisting (p=0.045).

Admission was only for burns (n=2) and falling (n=2) cases and only in Mena emergency hospital; otherwise, all trauma cases were discharged after management and able to complete their rituals - no deaths among the study sample.

## Discussion

In the Hajj season of the 1443 year of Hijri date (2022), the number of pilgrims is 899,353. The number of pilgrims outside KSA was 779,919 (87%), and the rest were citizens and residents from within [7]. This number is the biggest since the pandemic of the coronavirus disease 19 (COVID-19), which was around 2.5 million pilgrims in 2019 [8]. We found that the pattern of traumas and injuries in this Hajj season was similar to previous studies and seasons, with acceptable outcomes.

The demographics of the patients

The ages of patients are getting lower. In a study conducted in 2017, 40% of trauma patients were between 50 and 70 years, while in our study, 50% were between 33 and 48 years [9]. That may be because the Saudi government prioritizes those who perform Hajj for the first time and allows a low number of those who have performed the Hajj more than five years ago. Gender distribution is similar to previous studies, as they found that males with injuries are 70-80%, which is 73% in our study [9,10]. More education should be provided for males to avoid overcrowding and risky behaviors [11]. The Egyptians were the most common nationality, with a significant difference from the rest of the nationalities. In a previous study, the Saudis were the most common, followed by the Egyptians [9]. However, this may be due to the improvement of preparations before the Hajj by the campaigns responsible for the Saudi pilgrims. The Saudi health authorities and campaigns from Egypt need more effort to educate their pilgrims. Diabetes, followed by bronchial asthma, were the commonest comorbidities in our sample, while the same result was in a study conducted among pilgrims seeking medical services with unspecified diagnoses [10]. As in our study, comorbidity was not significantly associated with the type or cause of injuries.

The causes of injuries and trauma, and time-related issues

This study's current causes of traumas and injuries are similar to previous years. Although this study was conducted in the third year after COVID-19, and despite the relatively small numbers this year, overcrowding is still one of the trauma causes during Hajj, ensuring that overcrowding in mass gathering events is unavoidable [2,12].

Moreover, the causes differ between Arafat and Mena, as well as the time to reach the hospital and the times when injuries occur. The time required to reach the hospital is somewhat longer in Mena due to its large area [13]. In our study, most of the patients in Mena reach the hospital in 16 to 30 minutes, which is similar to a study conducted during the 2017 Hajj season [9]. Minimizing the duration will decrease further complications of traumas [4,14]. Therefore, educating the pilgrims about hospital distribution and free treatment availability may encourage them to go quickly and reduce the duration [3,7]. It may also make a special path for ambulances or wheelchairs and golf cars, which might decrease the duration, and at the same time, pilgrims must be aware not to walk on this path to prevent RTA. In a study conducted in a specialist hospital in Makkah during Hajj, 42% came from Mena mostly arrived between 6 and 12 pm, like our study [2]. In general, the reason for the occurrence of traumas and injuries at specific periods and not another in both areas is related to the fact that the rituals are specific at that time only. Also, the distance required and the place available for walking when performing the rituals differ in the two areas, as it is larger and longer in Mena and limited in a narrower place in Arafat. Likewise, specific rituals such as stoning the Jamarat in Mena or standing on Mount Arafa in the Arafat area affect the causes of trauma in both areas [13]. Educating the pilgrims generally accordingly is necessary to avoid such injuries.

Falling and foot twisting were the commonest causes of injuries in this study, which is similar to previous studies [9,12]. Foot twisting or ankle sprain was significantly higher in Arafat. Foot twisting and falling can lead to each other [15]. Therefore, falling and foot twisting was higher in Arafat, and more attention to managing the pilgrims in the Arafat area is needed.

Similarly, some causes disappear between the two areas. For example, burns from boiling water are present only in Mena, where the pilgrims spend most of their time in camps and housing, unlike Arafat, where all the sunburns are there since the pilgrims spend the day walking and standing in the sun for prayer. After all, boiling water burns were more common in the home and workplace in an earlier season of Hajj [2]. However, in a study conducted in 2011, the proportion of burns by boiling water and fire was 0.4% of traumas, while in our study, the proportion of burns by boiling water and sun was 0.2% [12]. Stressing not to use boiling water except in designated places is an excellent way to prevent this in the future. Fractures decreased in this season (5%) compared to the 2017 season, which was 28% in a study conducted in one hospital in Mena [9].

The type and site of injuries and trauma

Tissue contusion was the commonest type of injury in previous seasons [9,12]. In this study, the commonest type was cut wounds (50%), and the tissue contusion was 14%. This result may indicate better management of mass gathering, as tissue contusion is usually due to a force greater than the force causing superficial wounds. However, this study did not intend to differentiate between superficial and deep wounds; therefore, this result should be taken cautiously.

Blisters or friction blisters associated with walking are quite high in Mena. Friction blisters are preventable [16,17]. Avoid both; complications such as infection and hospital load are highly recommended and might be easily established. It could be done by educating pilgrims to wear appropriate shoes, not walk barefoot, and teaching them about first aid and when to go to the hospital [16]. However, diabetic patients had no blisters, which might indicate an appropriate level of awareness regarding using appropriate shoes, unlike what was reported in previous seasons of Hajj [6,18].

As in another study, the abdominally injured cases almost always had multisite trauma, and the cause was usually RTA [2]. Blunt abdominal injury is the commonest type in Hajj, as it is due to RTA, falling, and foot twisting. Blunt abdominal injuries are often missing and need more attention during hospital examinations [19].

The outcome of injuries and trauma

Although the situation was unfavorable in previous seasons of Hajj [20], it might be said that the organization of Hajj 1443H (2022) and hospitals' readiness to receive cases of traumas is improved. Outcomes are better as almost all patients were discharged (98%), and admission was not in intensive care or required major surgeries. In the study of the Saudi field epidemiology training program in the Hajj season of 1438H (2017) in Mena general hospital, the proportion of discharged trauma patients was 91% [9].

Limitations and future study recommendations

This study has a few limitations. Mena is covered medically by three hospitals. We decided to cover trauma cases in Emergency Mena Hospital, which receives most of what is considered an emergency, but the other hospitals also received part of trauma cases. Same in Arafat, other hospitals should be covered. A larger sample that includes all hospitals is needed in future studies. Some language barriers led to unfilled data, which was excluded from the analysis. There is a lack of studies regarding trauma and injuries in Hajj, so discussing the results may not be superior. Furthermore, this study is cross-sectional; thus, the future recommendation is to conduct case-control studies or qualitative studies to determine the predictors of traumas, investigate the relationship between the causes and outcome, and assess the implemented control measures. It is preferable to conduct multiple studies in the future in each season of Hajj, to recognize changes in the pattern [3].

## Conclusions

The Hajj season of 1443 H (2022) has a similar trauma pattern and improved outcomes compared to previous seasons. Discovering and digging into the causes of traumas and injuries should be optimized in future research for better control and customized prevention measures. Establishing new and remodeling current prevention measures is recommended for more control.
